# Autophagy is highly targeted among host comparative proteomes during infection with different virulent RABV strains

**DOI:** 10.18632/oncotarget.15184

**Published:** 2017-02-08

**Authors:** Ling Li, Hongli Jin, Hualei Wang, Zengguo Cao, Na Feng, Jianzhong Wang, Yongkun Zhao, Xuexing Zheng, Pengfei Hou, Nan Li, Hang Chi, Pei Huang, Cuicui Jiao, Qian Li, Lina Wang, Tiecheng Wang, Weiyang Sun, Yuwei Gao, Changchun Tu, Guixue Hu, Songtao Yang, Xianzhu Xia

**Affiliations:** ^1^ Department of Preventive Veterinary Medicine, College of Veterinary Medicine, Jilin University, Changchun, China; ^2^ Key Laboratory of Jilin Province for Zoonosis Prevention and Control, Institute of Military Veterinary, Academy of Military Medical Sciences, Changchun, China; ^3^ Department of Preventive Veterinary Medicine, College of Animal Science and Technology, Jilin Agricultural University, Changchun, China; ^4^ Jiangsu Co-innovation Center for Prevention and Control of Important Animal Infectious Disease and Zoonoses, Yangzhou, China; ^5^ School of Public Health, Shandong University, Jinan, China

**Keywords:** rabies virus, proteome, proteomics analysis, autophagy, differential virulence

## Abstract

Rabies virus (RABV) is a neurotropic virus that causes serious disease in humans and animals worldwide. It has been reported that different RABV strains can result in divergent prognoses in animal model. To identify host factors that affect different infection processes, a kinetic analysis of host proteome alterations in mouse brains infected with different virulent RABV strains was performed using isobaric tags for a relative and absolute quantification (iTRAQ)-liquid chromatography-tandem mass spectrometry (LC-MS/MS) proteomics approach, and this analysis identified 147 differentially expressed proteins (DEPs) between the pathogenic challenge virus standard (CVS)-11 strain and the attenuated SRV9 strain. Bioinformatics analyses of these DEPs revealed that autophagy and several pathways associated with autophagy, such as mammalian target of rapamycin (mTOR) signaling, p70S6K signaling, nuclear factor erythroid 2-related factor 2 (NRF2)-mediated oxidative stress and superoxide radical degradation, were dysregulated. Validation of the proteomic data showed that attenuated SRV9 induced more autophagosome accumulation than CVS-11 in an *in vitro* model. Our findings provide new insights into the pathogenesis of RABV and encourage further studies on this topic.

## INTRODUCTION

Rabies is a public health threat worldwide, and the pathogen, rabies virus (RABV), has high neurotropism and belongs to the genus *Lyssavirus* in the family Rhabdoviridae [1–3]. RABV infects all warm-blood animals and results in severe neurological symptoms with a mortality rate of approximately 100% [4]. Although rabies has been feared for more than 4,000 years, this virus causes approximately 59,000 human deaths per year, particularly in Asia and Africa [5, 6]. The gaps in our current understanding of the pathogenetic mechanisms of RABV are important barriers for combating this ancient disease.

Attenuated RABV can be cleared from the central neural system (CNS) in animal models, but pathogenic RABV uses stealth to replicate and spread without exposure and elimination. Efforts to explore the underlying mechanisms have been reported, and several research groups have conducted multi-omics studies, including transcriptomics, microRNomics, and proteomics analyses, to elucidate the major host factors associated with the pathogenesis of different strains [7–11]. Zhao et al. performed a microarray analysis to reveal the different transcript and microRNA profiles of mouse brains infected with street or laboratory-fixed RABV [9, 10, 12, 13]. Through two-dimensional electrophoresis, Hu et al. demonstrated that the overexpression of certain cytoskeletal proteins in neuroblastoma (NA) cells is positively correlated with the virulence of RABV [11]. Fu et al. analyzed gene expression profiles and showed that attenuated RABV triggers an extensive inflammatory response in mouse brains, whereas pathogenic RABV scarcely induces this response [7]. Subsequently, these authors demonstrated different immune responses after wild-type (wt) RABV and attenuated RABV infection and revealed a single divergent mechanism through which attenuated RABV triggers a high level of virus-neutralizing antibodies (VNA) in both serum and cerebrospinal fluid (CSF) after intrathecal (IT) infection, whereas wt RABV does not elicit VNA, and all infected dogs succumbed to rabies [14].

Alterations in the protein profiles of hosts with different virulent RABV infections might be responsible for the divergent prognoses. To batch-filter host factors that might affect infection, a kinetic analysis of alterations in the host proteome of mouse brains infected with RABV strains with differential virulence was conducted using isobaric tags for a relative and absolute quantification (iTRAQ)-liquid chromatography-tandem mass spectrometry (LC-MS/MS) proteomics approach. This approach was selected based on its superior performance for simultaneous comparisons of multiple samples with wide dynamic ranges of protein abundance [15, 16]. This proteomics analysis revealed that compared with mock infection, the attenuated vaccine strain SRV9 induced more in-depth global dysregulation than the laboratory-fixed RABV challenge virus standard (CVS)-11 strain. Because the primary objective of this comparative proteomics analysis was to screen the major factors affecting pathogenicity, we subsequently performed a bioinformatics analysis of the differentially expressed proteins (DEPs) between the CVS-11 and SRV9 groups using the QIAGEN Ingenuity Pathway Analysis (IPA) platform. The results showed that autophagy and autophagy-associated pathways, including mammalian target of rapamycin (mTOR) signaling, mitochondrial dysfunction, nuclear factor erythroid 2-related factor 2 (NRF2)-mediated oxidative stress response, and superoxide radical degradation, were highly enriched when compared the two infected groups. Based on these analyses, we validated that attenuated SRV9-infected NA cells presented more increased autophagosome accumulation compared with pathogenic CVS-11-infected cells. These results provide new insights into RABV pathogenesis and encourage further studies on this topic.

## RESULTS

### SRV9 is less pathogenic than CVS-11 and can be eliminated from the CNS

**Figure 1:**
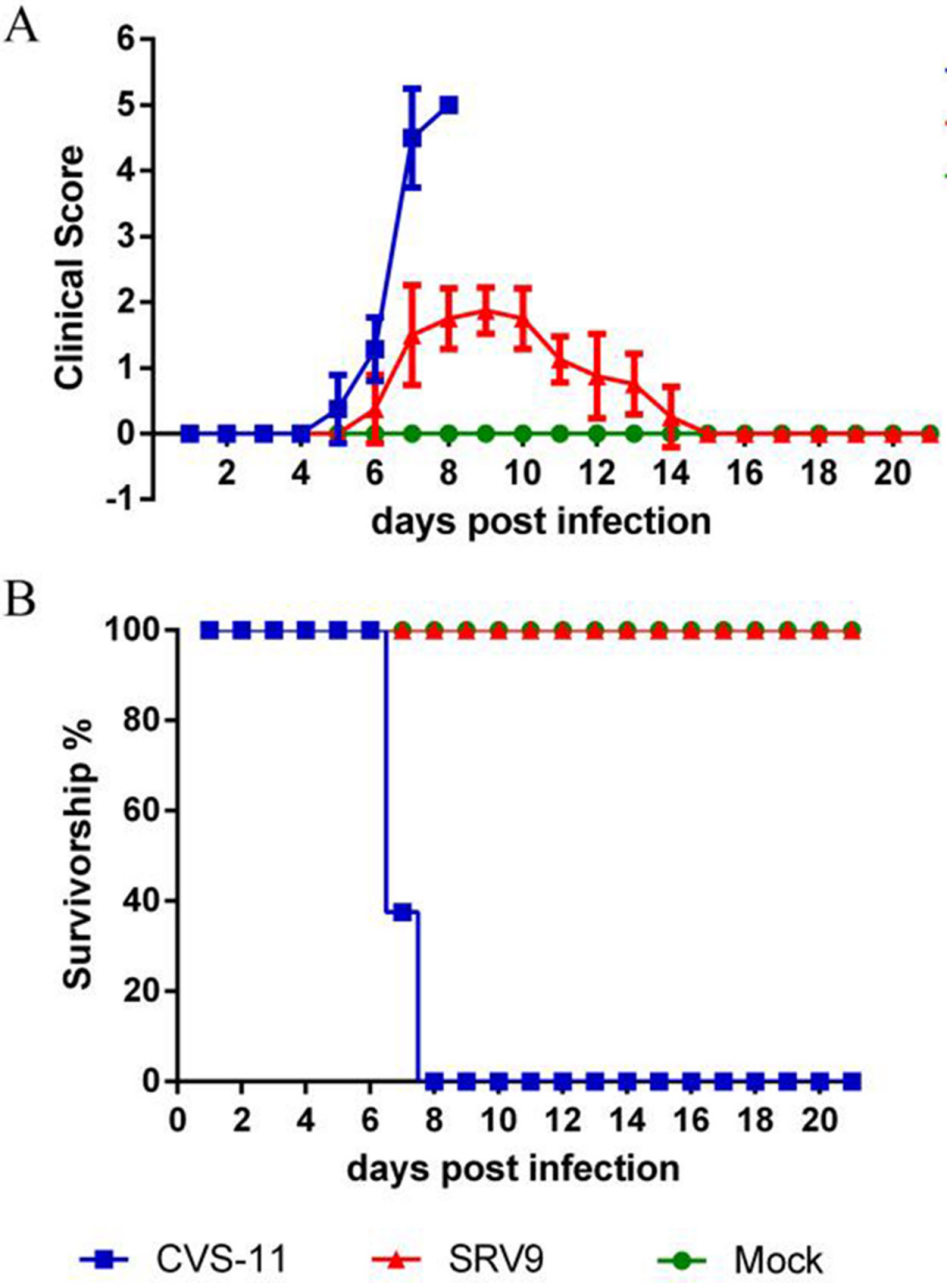
SRV9 is less pathogenic to mice than CVS-11 after i.c. inoculation Mice (n=8) were infected (i.c.) with a TCID_50_ of CVS-11 or SRV9 equal to 10^5^ or an equal volume (30 μl) of DMEM as a control. The mice were observed for clinical signs A. and survival B. for 21 days.

Mice infected with CVS-11 developed their initial clinical symptoms at 5 days post infection (dpi) and showed rapid deterioration resulting in paralysis. The mice were dead at 7 to 8 dpi. In comparison, the appearance of SRV9-induced rabies signs was delayed to 6 dpi, and huddled behavior and ruffled fur being apparent by 9 dpi. The mice infected with SRV9 showed gradual recovery between 10 and 14 dpi and presented no visible clinical signs at 15 dpi (Figure 1).

To confirm whether the mice were successfully infected with RABV and to determine which time points were suitable for the proteomics analysis, the genomic RNA and virus titers in the mouse brains were detected at 1, 4, and 7 dpi. As shown in Table 1, traces of genomic RNA were detected in all brains, and the virus was identified in two of three brains per group at 1 dpi. Both the genomic RNA and virus titers were higher in the mice infected with CVS-11 than in those infected with SRV9 at 4 dpi. At 7 dpi, a higher viral load and higher levels of genomic RNA were observed in the CVS-11-infected group, but no virus and only traces of genomic RNA were detected in the attenuated SRV9 group, indicating that attenuated RABV was almost cleared from the CNS at this time point.

**Table 1:** RABV genomic RNA and virus titers in the brains of mice infected with RABV

Days post infection	Genomic RNA (log(copy number / μg total RNA))		Virus titers (log(TCID_50_ / g tissue))	
	CVS-11 infection	SRV9 infection	CVS-11 infection	SRV9 infection
1	2.85, 1.33, 2.70	2.82, 2.87, 2.12	2.67, -^a^, 2.65	3.48, 3.50, -
4	4.73, 4.92, 4.98	3.37, 3.54, 3.75	5.40, 5.58, 4.78	4.55, 4.10, 4.41
7	3.80, 4.70, 5.07	1.65, 2.39, 1.73	5.44, 5.77, 5.73	-, -, -

^a^ No virus was detected.

Based on these results, we selected the time points of 1, 4, and 7 dpi for our proteomics analysis as representative times for the early stage of infection (1 dpi), the initial appearance of body weight loss (data not shown), clinical symptoms and appropriate viral load (4 dpi), and either death (of the pathogenic CVS-11-infected mice) or viral clearance stage (of the attenuated SRV9-infected group; 7 dpi), respectively.

### Global protein profile of mouse brains infected with RABV

Protein extracts of CVS-11- and SRV9-infected mouse brains obtained at 1, 4, and 7 dpi combined with protein extracts from mock-infected mouse brains were subjected to iTRAQ coupled with LC-MS/MS analysis. In total, 2,285 non-redundant proteins were identified. A significantly up- or down regulated protein was determined when its |fold change, (FC)|≥1.3 and p≤0.05. Using the protein profile of a mock-infected mouse brain as the background, 90 non-redundant DEPs_vs.m_, consisting of 10, 25 and 49 upregulated proteins and 13, 10, and 21 downregulated proteins at 1, 4, and 7 dpi, respectively, were identified in the pathogenic CVS-11-infected mouse brains. In addition, the attenuated SRV9 strain induced more in-depth global dysregulation, and 227 non-redundant DEPs_vs.m_, including 3, 28, and 176 upregulated proteins and 22, 12, and 23 downregulated proteins at 1, 4, and 7 dpi, respectively, were identified. A complete list of the dysregulated proteins is shown in Supplementary Table 1.

**Figure 2:**
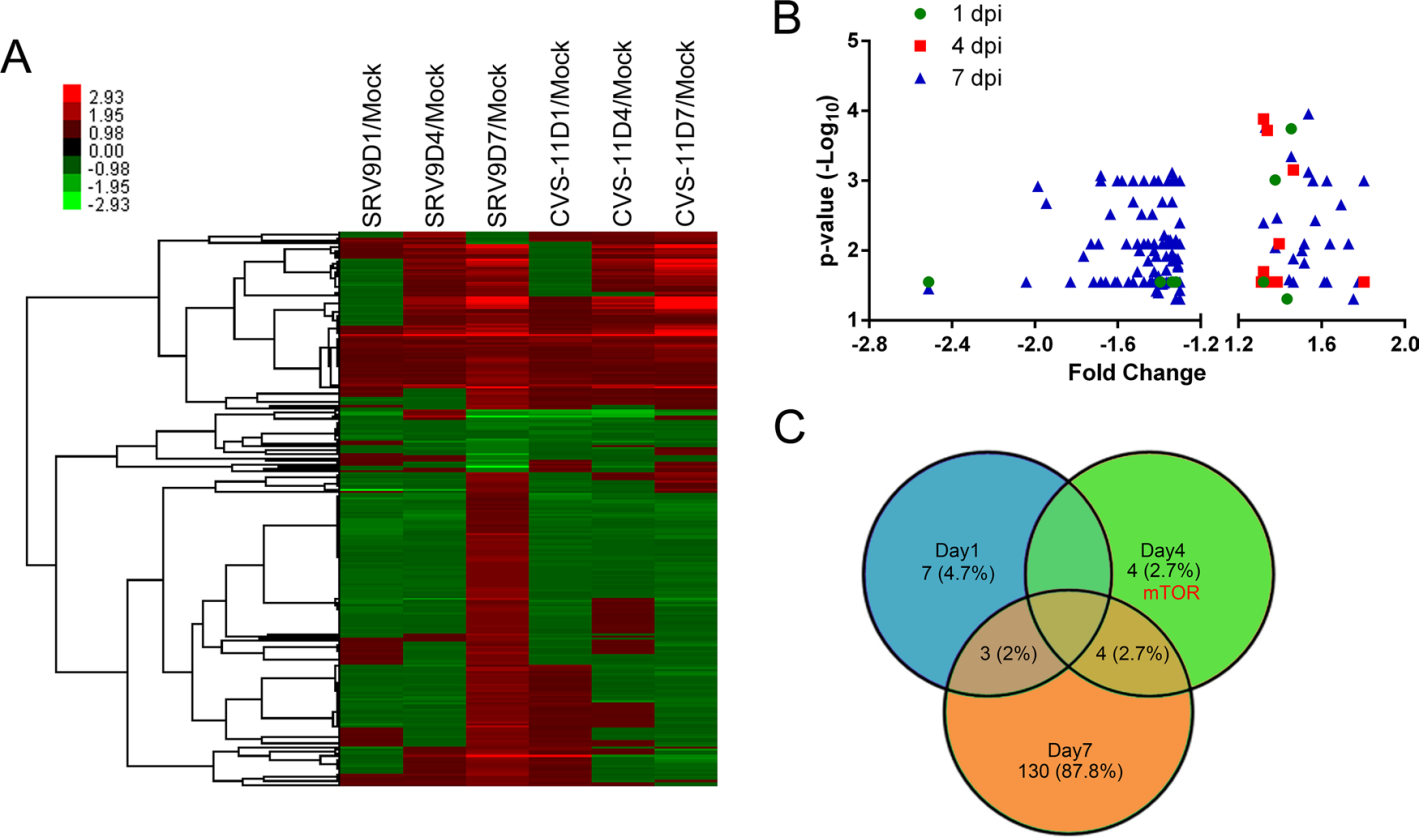
Patterns of differentially expressed host proteins at three time points in mouse brains infected with the CVS-11 or SRV9 strain of RABV **A**. Attenuated SRV9 triggers a more in-depth response to infection than pathogenic CVS-11. A heatmap of hierarchical clustering using the average linkage method demonstrated 265 non-redundant DEPs_vs.m_ at the three time points. The color bar represents the degree of up- and downregulation. **B**. Volcano plots of DEPs between the CVS-11- and SRV9-infected groups show the FC (x-axis) and significance (y-axis) at three time points. The plots indicate that the highest amount of dysregulation was observed at 7 dpi, and a majority of the DEPs were overexpressed in the SRV9 group compared with the CVS-11 group. **C**. Venn diagram depicting the relationship of the DEPs between the CVS-11- and SRV9-infected groups at the three time points. The corresponding percentage of host proteins (shown in brackets) that are significantly differentially regulated in each category is indicated.

After eliminating repetition, 265 DEPs_vs.m_ were identified compared with mock-infected mouse brains. Figure 2A shows the results of the hierarchical cluster analysis of these DEPs_vs.m_, and the results show that the majority of these proteins presented similar variation tendencies at 1 and 4 dpi, corresponding to the similar clinical symptoms observed in the two groups at these stages of infection. However, at 7 dpi, more than half of the DEPs_vs.m_ were overexpressed in the attenuated SRV9-infected mouse brains but downregulated in the pathogenic CVS-11-infected group. These proteins might be responsible for the different clinical symptoms observed between the two groups. A comprehensive comparison of the proteome profiles showed that some of the DEPs_vs.m_ were not significantly different between the SRV9- and CVS-11-infected groups. Because the major objective of the present study was to determine which factors influence the pathogenicity of different strains of RABV, we focused on proteins that exhibit different expression levels between the two infected groups and subjected these to a bioinformatics analysis.

Using the global protein profile of SRV9-infected mouse brains as the background, 148 DEPs_vs.s_ (hereafter referred to as DEPs unless otherwise specified), consisting of 10 upregulated proteins and no downregulated proteins at 1 dpi, 4 upregulated and 4 downregulated proteins at 4 dpi, and 28 upregulated and 109 downregulated proteins at 7 dpi, were identified in the CVS-11-infected group. The volcano plots and intersection of the DEPs are illustrated in Figures 2B and 2C. The results showed that only a few proteins were significantly differentially expressed at 1 and 4 dpi, and these corresponded to the clinical observations, i.e., no obvious differences were found between the two groups at these time points. The highest number of dysregulated proteins was observed at 7 dpi, and most of these were downregulated in the CVS-11 group compared with the SRV9 group. In addition, in this time point the infected mice also showed clearly distinct clinical signs leading to different prognoses.

### Functional enrichment and network analysis of the CVS-11- *vs*. SRV9-infected mouse brains

To investigate the biological functions that are potentially dysregulated upon infection with RABV strains of different virulence, the list of DEPs was imported into the IPA platform, and a core analysis was performed. The IPA generated two algorithms for evaluating the identified biological functions: the p-value, which is used to determine the pertinence of a group of proteins belonging to specific functional categories, and the z-score, which is used to predict whether the functions were activated or inhibited based on the expression value and current knowledge [17].

**Figure 3:**
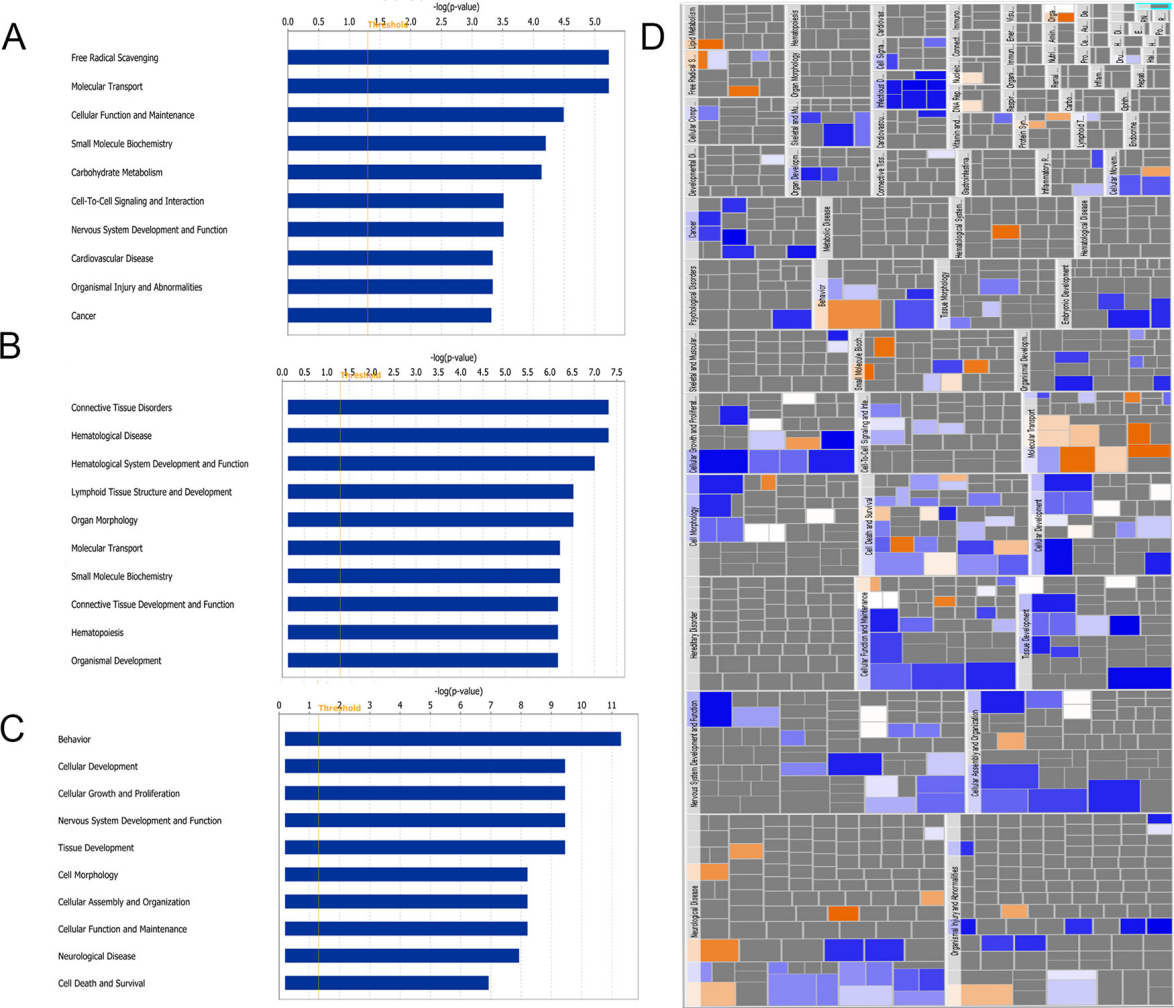
Functional characterization of DEPs identified at 1 A. 4 B. and 7 C and D. dpi A, B, and C show the top 10 disease and function terms at each time point. D shows the heatmap of the diseases and functions regulated at 7 dpi. Large rectangles represent broad categories of biological functions and diseases, whereas small rectangles represent individual terms defined in the IPA database. The area of each rectangle is proportional to the dysregulation of each term (-log10 of the p-value), and the colors represent the predicted activation (orange) or inhibition (blue) of each specific term.

Figures 3A to 3C show the histograms of the top 10 functions associated with DEPs at each time point. The top affected functions at 1 dpi included free radical scavenging, molecular transport, cellular function and maintenance, and small molecular biochemistry (Figure 3A). The top functions at 4 dpi included connective tissue disorders, hematological disease, hematological system development and function, lymphoid tissue structure and development and organ morphology (Figure 3B), whereas those identified at 7 dpi were behavior, cellular development, cellular growth and proliferation, and nervous system development and function (Figure 3C). A heatmap was also constructed to exhibit the pertinence (p-value, indicated as the area of each rectangle) and the prediction of activation or inhibition (z-score, indicated by the color of the rectangle) of the functions associated with the DEPs at 7 dpi. As shown in Figure 3D, the affected function categories include neurological disease, organismal injury and abnormalities, and nervous system development and function. Moreover, the functions that were predicted to be significantly activated included secretion of lipid (z-score = 2.19), and the functions and diseases that were predicted to be significantly inhibited were viral infection (z-score = -3.601), generation of cells (z-score = -3.415), cancer (z-score = -2.928), development of neurons (z-score = -2.6), and infection by RNA virus (z-score = -2.567). These results provide a broad overview of bioinformatically predicted cellular-level dysregulation that might potentially be involved in the pathogenesis of different RABV strains. The details of the biological functions and diseases analyzed by IPA are available in Supplementary Table 2.

**Figure 4:**
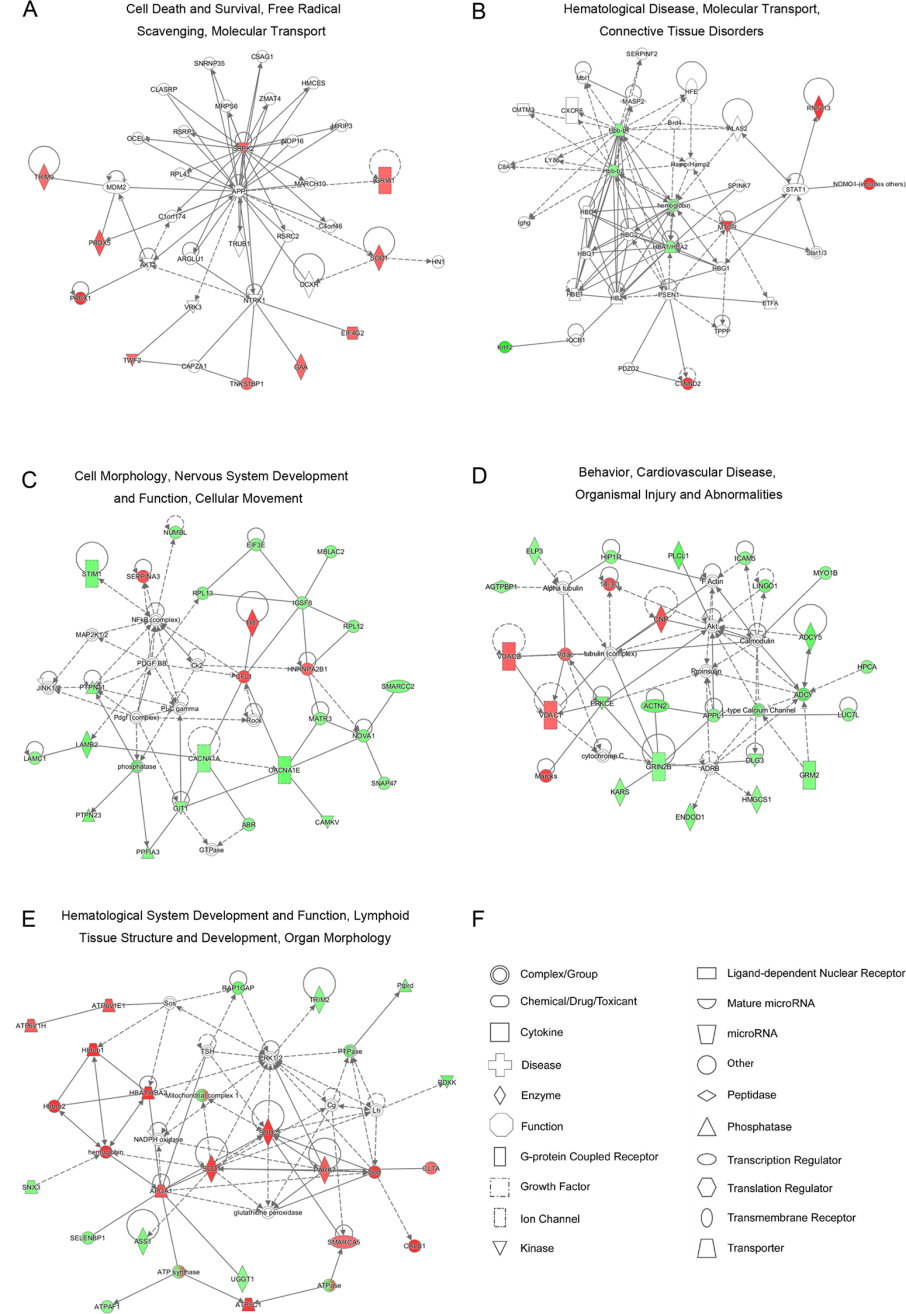
Significant protein-protein networks of DEPs identified between pathogenic CVS-11 and attenuated SRV9 infections **A-E**. Red represents the upregulated proteins, green represents the downregulated proteins, and white represents proteins belonging to the network but not identified in this proteome. The color depth represents the magnitude of the alterations in protein expression. The solid and dashed lines indicate direct and indirect interactions, respectively. **F**. Different shapes represent unique molecular types.

IPA provided a systematic overview of protein-protein/RNA/small molecule interactions and generated a score to evaluate the relevance of the submitted molecules to the network. Figure 4 and Supplementary Table 3 show the mapped networks. Because the small number of DEPs were identified at 1 and 4 dpi, only a single credible network was established at each time point: “cell death and survival, free radical scavenging, molecular transport” (10 molecules; score 29; Figure 4A) at 1 dpi and “hematological disease, molecular transport, connective tissue disorders” (8 molecules; score 24; Figure 4B) at 4 dpi. Notably, free radical scavenging associated with oxidative stress emerged during the early stage of RABV infection. To validate the oxidative stress observed during RABV infection, reactive oxygen species (ROS) generation was detected using the fluorescent dye 6-carboxy-2′, 7′-dichlorodihydrofluorescein diacetate (DCFH-DA, Beyotime Biotechnology, Jiangsu, China). As shown in Supplementary Figure 1, similar levels of ROS generation were observed in the RABV- and mock-infected NA cells at 12 and 24 hours post infection (hpi). A rapidly increasing production of ROS was observed at 36 hpi in the CVS-11 group, and this high production level was maintained through 48 hpi. In comparison, the SRV9-induced increase in ROS production was delayed to 48 hpi. Moreover, based on the abundantly identified DEPs, multiple networks were significantly targeted at 7 dpi, and the top three networks were “cell morphology, nervous system development and function, cellular movement” (25 molecules; score 52; Figure 4C), “behavior, cardiovascular disease, organismal injury and abnormalities” (23 molecules; score 46; Figure 4D), and “hematological system development and function, lymphoid tissue structure and development, organ morphology” (22 molecules; score 44; Figure 4E). These networks reflect the outward manifestation of rabies (such as behavior, organismal injury and abnormalities) and potential intrinsic causes of this disease (involving physiological parameters such as hematological system development and function, lymphoid tissue structure and development).

### Autophagy and autophagy-associated pathways are highly targeted in CVS-11- *vs*. SRV9-infected mouse brains

**Figure 5:**
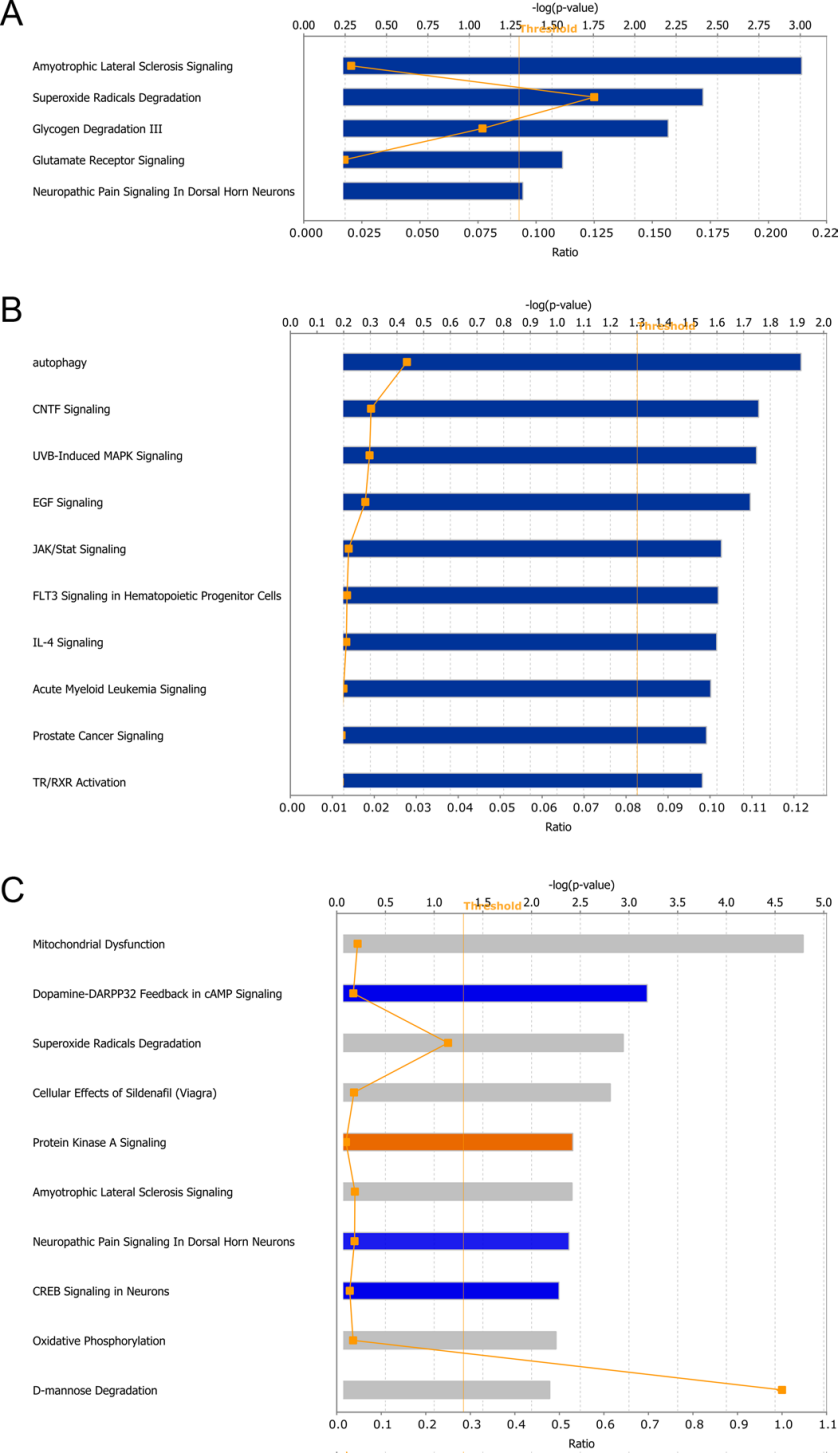
Canonical pathways predicted modulated between different virulent RABV infections **A, B** and **C**. represents the identified pathways at 1, 4 and 7 dpi, respectively. The broken lines with rectangle represent the ration of DEPs in corresponding pathway. The color in C represents the bioinformatics results predicting pathway activity; orange: predicted activation; blue: predicted inhibition; and gray: no activity pattern available.

The IPA canonical pathway analysis provides a statistical evaluation of the regulated pathways based on information of the altered proteins. The algorithms used for evaluating functional enrichment were also applied in this module. At 1 dpi, only five canonical pathways were significantly dysregulated: amyotrophic lateral sclerosis signaling, superoxide radicals degradation, glycogen degradation III, glutamate receptor signaling, and neuropathic pain signaling in dorsal horn neurons (Figure 5A). At 4 dpi, 22 pathways were significantly dysregulated, and the top 5 canonical pathways were autophagy, CNTF signaling, UVB-induced MAPK signaling, EGF signaling, and JAK/stat signaling. Figure 5B shows the top 10 altered pathways. At 7 dpi, 47 canonical pathways were significantly modulated, and the top 10 are ranked in Figure 5C according to p-value. The most affected pathways included mitochondrial dysfunction, dopamine-DARPP32 feedback in cAMP signaling, superoxide radicals degradation, cellular effects of sildenafil (Viagra), and protein kinase A signaling. Five canonical pathways were inhibited, as determined through an evaluation of the z-score: dopamine-DARPP32 feedback in cAMP signaling (z-score = -2.24), CREB signaling in neurons (z-score = -2.24), cardiac hypertrophy signaling (z-score = -2.24), neuropathic pain signaling in dorsal horn neurons (z-score = -2.00) and synaptic long term potentiation (z-score = -2.00). Supplementary Table 4 shows the details of the dysregulated pathways.

**Figure 6:**
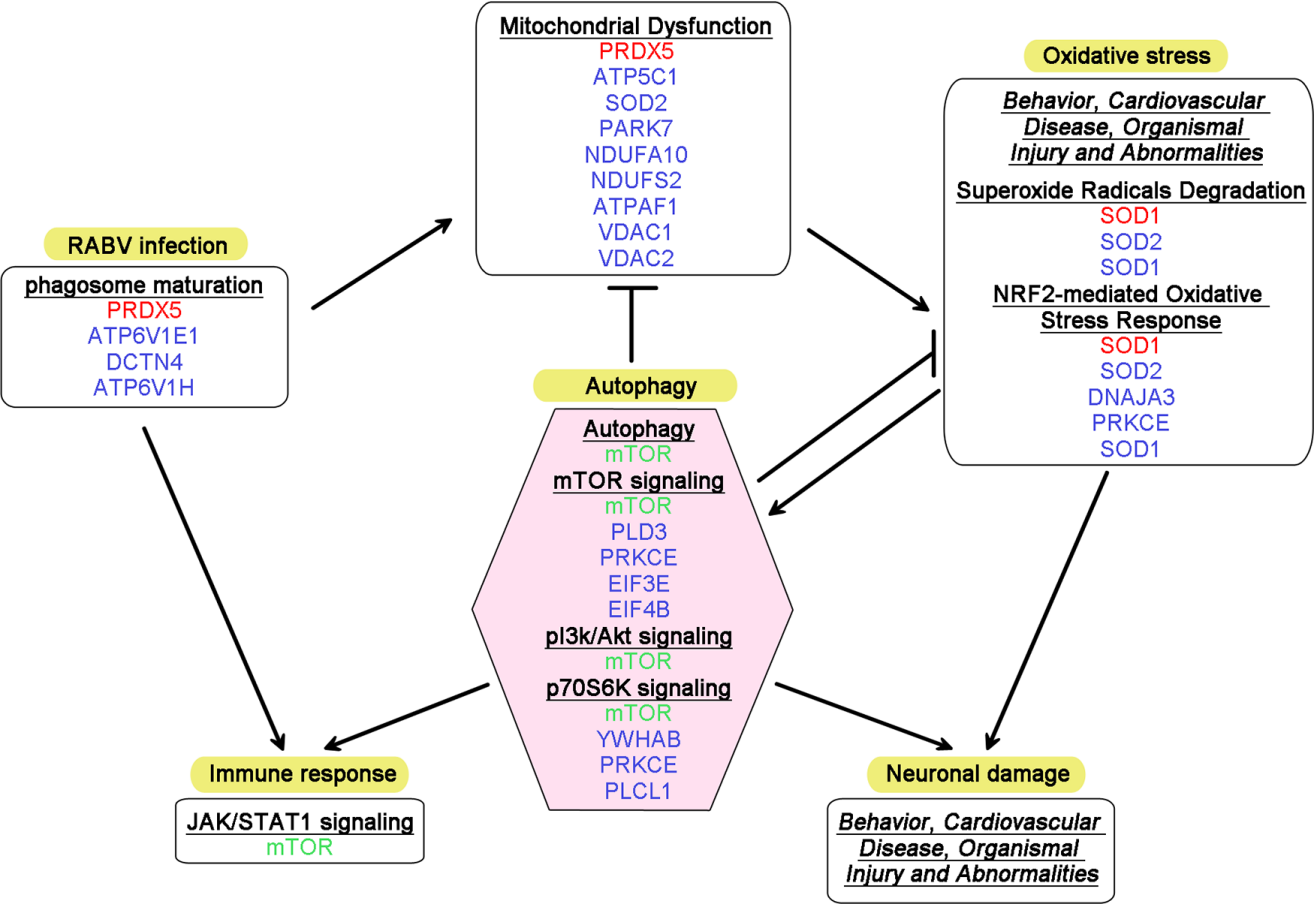
Diagram summarizing the altered canonical pathways and networks associated with autophagy in RABV infection DEPs identified at 1, 4 and 7 dpi are tinted with red, green and blue, respectively. The canonical pathways are shown in bold and underlined font, and the networks are shown in italic and underlined font. The arrows represent induction, and the other lines represent inhibition or remission.

The pathway analysis during the progression of rabies highlighted several terms associated with autophagy, including superoxide radicals degradation (-log(p-value) = 2.41), mitochondrial dysfunction (-log(p-value) = 1.10), and mTOR signaling (-log(p-value) = 1.06) at 1 dpi. Although only 8 DEPs were identified at 4 dpi, this time point remains interesting because of the crucial role of mTOR, one of the DEPs at this time point, in a number of biological pathways, particularly autophagy. The validation of mTOR signaling is shown in Supplementary Figure 2. At 7 dpi, pathways associated with autophagy, including mitochondrial dysfunction (-log(p-value) = 4.97), superoxide radical degradation (-log(p-value) = 2.94), mTOR signaling (-log(p-value) = 1.47), and p70S6K signaling (-log(p-value) = 1.37), were even more disordered, indicating that autophagy might participate in RABV infection. Figure 6 shows a speculative diagram of the pathways likely associated with autophagy during RABV infection.

### Attenuated SRV9 infection results in more autophagosome accumulation than pathogenic CVS-11 infection

Based on the information obtained from the comparative proteome analysis, autophagy was prominently targeted between pathogenic CVS-11 and attenuated SRV9 infections, indicating its possible involvement in RABV infection. Therefore, we subsequently validated whether the autophagy pathway was dysregulated during RABV infection using NA cells as an *in vitro* model.

**Figure 7:**
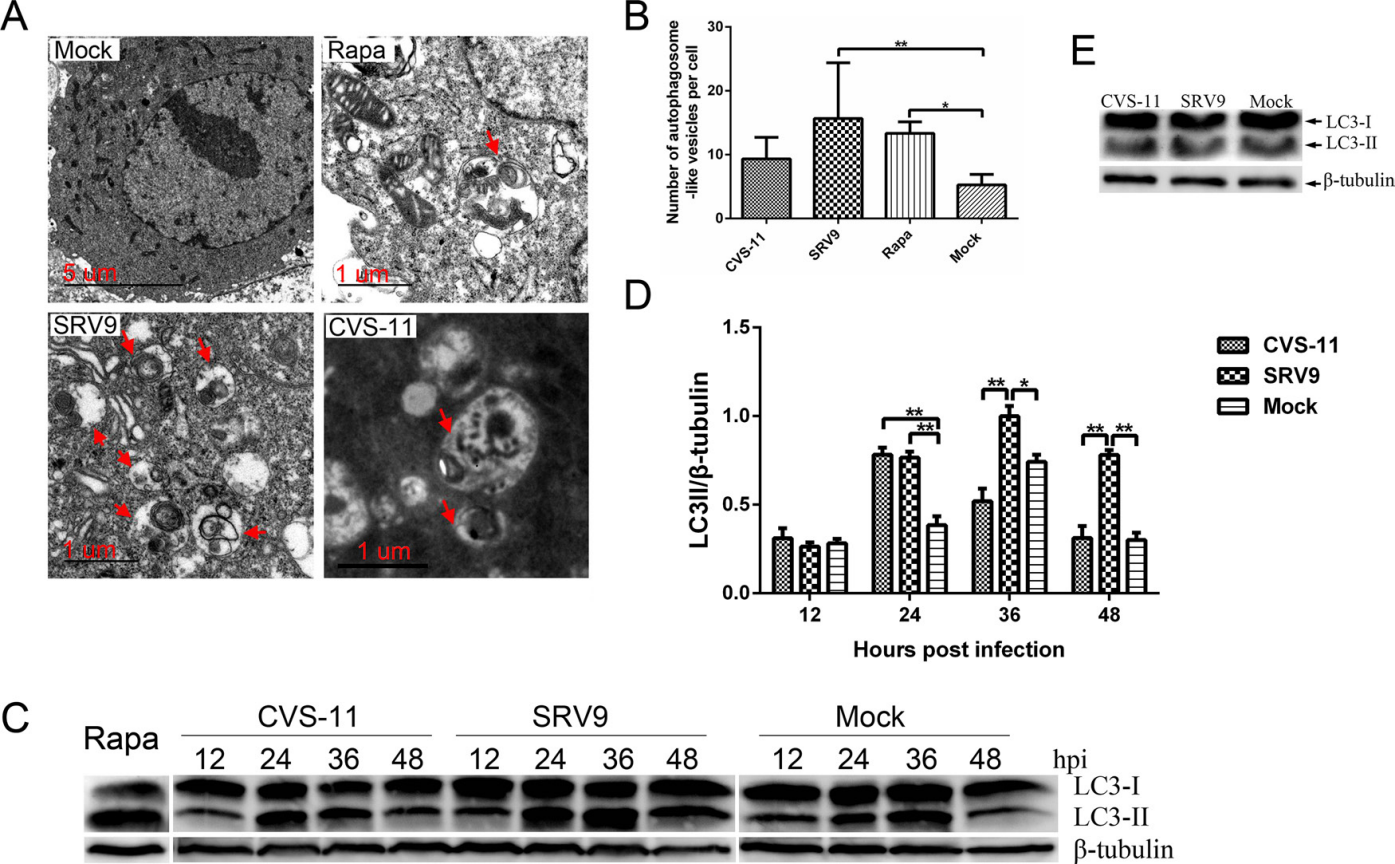
RABV infection induces autophagosome accumulation in NA cells **A**. TEM observation. NA cells were mock-infected or infected with the RABV CVS-11 or SRV9 strain at a MOI of 5 for 36 h. Cells treated with 100 nM rapamycin served as a positive control. A TEM analysis was subsequently performed. The arrows indicate autophagosome-like vesicles. **B**. Quantification of the autophagosome-like vesicles per cell. The average number of vesicles in each cell was obtained from at least 10 cells. **C**. Western blot analysis of the LC3-II expression levels. NA cells were mock-infected or infected with the RABV CVS-11 or SRV9 strain at a MOI of 5. The cells were harvested and lysed at the indicated time points, and the cell extracts were subsequently probed with an anti-LC3B antibody. β-tubulin was used as a protein loading control. Representative images from three replicates are shown. **D**. The LC3-II-to-β-tubulin intensity ratio was calculated. The data represent the means ± SD of three independent experiments. **E**. Western blot analysis of LC3-II levels in NA cells infected with UV-inactivated CVS-11 and SRV9. The results are representative of three independent experiments. The significance was analyzed through one-way ANOVA. *, p≤0.05; **, p≤0.01.

Mammalian autophagy was originally identified through electron microscopy in the late 1950s, and transmission electron microscopy (TEM) observations remains the standard method for detecting autophagosomes [18]. The ultrastructure of an autophagosome is described as a double- or single-membrane vesicle containing sequestered intracellular organelles. As shown in Figure 7A, an increased number of membrane vesicles was observed in NA cells infected with RABV, and undigested mitochondria were observed inside the vesicle. A similar ultrastructure was rarely observed in the mock-infected NA cells. A quantitative analysis revealed that the number of autophagosomes in SRV9- but not CVS-11-infected NA cells was significantly higher than that observed in the mock-infected groups (Figure 7B).

There is a good correlation between microtubule-associated protein 1 light chain 3 (LC3)-II (lipidated LC3) and the number of autophagosomes; hence, the expression level of LC3-II was detected by western blotting [19]. The LC3-II expression levels in both RABV-infected groups were the same as that observed in uninfected NA cells at 12 hpi. At 24 hpi, the LC3-II levels in both RABV-infected groups were significantly higher than those in the mock-infected group, whereas by 36 and 48 hpi, the levels in the pathogenic CVS-11- but not the SRV9-infected NA cells had decreased to those found in the mock-infected cells. Rapamycin-treated cells served as a positive control, indicating the overexpression of LC3-II (Figure 7C and 7D).

To determine whether viral replication is required for autophagy induction, NA cells were exposed to ultraviolet (UV)-inactivated RABV. No detectable conversion from LC3-I to LC3-II was observed, with no differences compared with the mock-infected group (Figure 7E). This result indicates that viral replication, and not simply the interaction between noninfectious virus particles and host cells, is essential for RABV-induced autophagosome accumulation.

Taken together, the results shown in Figure 7 indicate that the autophagy pathway is actually dysregulate during RABV infection, and SRV9 leads more autophagosome accumulation in NA cells than CVS-11.

## DISCUSSION

The balance between the host response and viral invasion largely determines the clinical outcome of a viral infection. Proteomics technology has been widely used for probing virus-host interactions, and several comparative proteomes have identified factors that affect the pathogenesis of strains of differential virulence or at different stages of virus infection [8, 17, 20, 21]. Here, iTRAQ combined with LC-MS/MS was applied to analyze the protein profiles of mouse brains infected with pathogenic CVS-11 and attenuated SRV9 strains of RABV to identify the host factors that affect the pathogenesis of RABV. The present study shows the attenuated SRV9 strain induced more in-depth global dysregulation than the pathogenic CVS-11 strain. A bioinformatics analysis revealed that the DEPs were highly targeted to autophagy and autophagy-related pathways, and subsequent analyses demonstrated that RABV infection increased the accumulation of autophagosomes, with differences in infection phases between CVS-11 and SRV9 strains. These results provide a comprehensive understanding of how the host manages RABV challenge and should provide new insights into the pathogenesis of RABV.

Several studies have reported differences in the pathogenesis of RABV strains with different levels of virulence. Yin et al. showed that RABV strains present differences in the induction of histopathological and ultrastructural changes in mouse brains and demonstrated that these differences were exhibited in terms of the degrees of cell apoptosis, neuronal dendrite damage, microglial activation and T lymphocyte infiltration [22]. Vaziri et al. demonstrated the divergence of peripheral immune responses to different strains of RABV with different pathogenicities using a two-dimensional comparative proteomics approach [23]. At the transcriptome level, Fu et al. demonstrated that attenuated RABV activates host innate immune responses, whereas pathogenic RABV invades the CNS [7]. Observations were then obtained with dogs to show that IT immunization of laboratory-attenuated RABV induces high levels of VNA at the periphery and in CSF while transiently enhancing the blood-brain barrier (BBB) permeability, whereas wt RABV does not show the capacity to induce VNA and specific antibodies against rabies viral glycoprotein, resulting in the death of the treated animals [14]. Due to the superior performance of the iTRAQ LC-MS/MS proteomics approach for the simultaneous comparison of multiple samples with wide dynamic ranges of protein abundance, an increasing number of studies have applied this method to explore the factors influencing the pathogenesis of virus strains with different degrees of virulence [15, 17, 21]. In the present study, we performed a comparative proteomics analysis of mouse brains infected with pathogenic CVS-11 and attenuated SRV9 strains of RABV and showed that the DEPs between the two groups were highly enriched in autophagy and related pathways. These indicate that autophagy might be involved in the progression of RABV infection.

Autophagy is an ancient, evolutionarily conserved intracellular degradation process for the removal of damaged organelles and protein aggregates from the cytoplasm [24]. Autophagy has become an intense field of research, reflecting its crucial role in homeostasis and its impact on antigen presentation, pathogen recognition and the connection between innate and adaptive immune responses [25–28]. Recently, an increasing number of studies have demonstrated that autophagy is involved in viral infection and affects the replication and/or pathogenicity of viruses [29–32]. Vesicular stomatitis virus (VSV), another member of Rhabdoviridae, could trigger autophagy in plasmacytoid dendritic cells (pDCs), and the formed autophagosomes could deliver viral nucleic acids to endosome-resident Toll-like receptor (TLR) 7 for IFN induction [33]. Subsequently, Shelly et al. demonstrated that VSV-triggered autophagy is an essential component of the host antiviral response in *Drosophila* [34]. However, autophagy is a double-edged sword, and many viruses have evolved strategies to evade, subvert, or even exploit autophagy. Ding et al. revealed that Newcastle disease virus (NDV) could trigger autophagy *in vitro* and *in vivo*, resulting in enhanced virus replication, and treatment with autophagy inhibitors reduces NDV titers in the lungs of chickens and ameliorates NDV-induced lung and intestine injury, thereby increasing the survival rate of NDV-infected chickens [35, 36]. Moreover, autophagy might play a divergent role in the differential virulence or serotypes of the same virus. Both dengue virus (DENV)2 and DENV3 infection induce and require autophagy; however, the replication of DENV2 is increased upon treatment with an inhibitor of autophagosome-lysosome fusion, whereas treatment with a lysosomal fusion inhibitor decreases DENV3 replication [37–39]. However, not every viral infection is involved in the autophagy pathway. Vandergaast et al. revealed that West Nile virus (WNV), in contrast to several other members of *Flaviviridae*, such as Japanese encephalitis virus (JEV), DENV, hepatitis C virus (HCV) and classical swine fever virus (CSFV), does not interact with the autophagy pathway [30, 40–44]. In this study, we found that the autophagy pathway was dysregulated during RABV infection and that SRV9 leads more autophagosome accumulation in the infected cells than CVS-11 (Figures 6 and 7). This information might contribute to our current understanding of the pathogenesis of RABV and encourage further studies on this topic.

Oxidative stress is a common factor that induces autophagy, and in return, autophagy plays a protective role in cells resistant to oxidative damage [45, 46]. *In vitro* studies have found that RABV infection induces oxidative stress in response to mitochondrial dysfunction [47, 48]. Kammouni et al. demonstrated a higher rate of ROS generation in CVS-infected neurons in the presence of mitochondrial substrates and inhibitors, suggesting that RABV infection induces mitochondrial dysfunction leading to increased ROS generation and oxidative stress [49]. Subsequently, Kammouni et al. demonstrated that the phosphoprotein of RABV, particularly amino acids 139-172, interacts with complex I in mitochondria, causing mitochondrial dysfunction and increased generation of ROS and oxidative stress [49]. The present proteomics analysis revealed that several terms associated with oxidative stress, such as mitochondrial dysfunction, superoxide radical degradation, and NRF2-mediated oxidative stress response, were dysregulated. The validation of ROS generation showed that the increase in ROS generation was delayed in SRV9- compared with CVS-11-infected cells (Supplementary Figure 1). Together with Figure 7C and 7D, we speculate that autophagy might play a protective role in the resistance of NA cells to RABV-induced oxidative stress, but the crosstalk between autophagy and oxidative stress during RABV infection requires further study.

In conclusion, this study constitutes the first kinetic comparative proteomics investigation to comprehensively analyze and explore the differences in the whole host response to infection with RABV strains with differential pathogenicities. The results revealed that many factors associated with autophagy differed between the two RABV-infected groups, and subsequent studies demonstrated that the degrees of virus-induced autophagosome accumulation were different between the two groups. Moreover, the pathways through which RABV trigger autophagy and the role of autophagy, antiviral or proviral, in RABV infection will be the major issues addressed in future studies. In conclusion, the present study provides a novel approach for elucidating the pathogenesis of RABV.

## MATERIALS AND METHODS

### Ethics statement

The animal experiments performed in the present study were approved by the Animal Care and Use Committee of the Chinese People's Liberation Army (No. SYXK2009-045). The animals received humane care, which was managed according to the rules of 3R (i.e., reduction, refinement and replacement). All efforts to minimize mouse suffering were considered and implemented when possible.

### Animals, cells and viruses

Female BALB/c mice (6 to 8 weeks old) were purchased from the Changchun Institute of Biological Products, China, and housed in temperature- and light-controlled quarters with free access to water and food. Two RABV strains were used in the present study to explore the discrepancies in the host response against RABV strains with different degrees of virulence: the CVS-11 strain, which exhibits high pathogenicity, and the SRV9 strain, an attenuated virus derived from the SAD strain through plaque purification in baby hamster kidney (BHK) cells [50]. The virus stocks were grown in NA cells of A/J mouse origin in Dulbecco's modified Eagle's medium (DMEM; Gibco, CA, USA) containing 2% fetal bovine serum (FBS; Gibco, CA, USA). The viruses were titered as previously described [51] and subsequently aliquoted and stored at -80°C until further use. UV-inactivated RABV was obtained after exposing the viral suspension to UV light for 1 h, and the absence of infectivity was confirmed through detection of the viral titer followed by aliquoting and storage at -80°C until further use.

### Viral infection

The mice were infected with a 50% tissue cultured infective dose (TCID_50_) of each virus (CVS-11 or SRV9) equal to 10^5^ in a volume of 30 μl through the intracerebral (i.c.) route, and sham-infected mice were used as controls. The mice were observed daily for the development of rabies, and clinical symptoms were evaluated and scored as previously described [52]. At 1, 4 and 7 dpi, five mice per group were sacrificed. The brains were collected, flash-frozen and stored at -80°C. Two mouse brains per group were used for the iTRAQ LC-MS/MS proteomics analysis, and the other three were used to determine the RABV genomic RNA and virus titers as previously described [51].

For the detection of autophagosome accumulation, monolayers of NA cells were infected at a multiplicity of infection (MOI) of 5. After 1 h of adsorption, the inoculum was removed, and the cells were washed three times to rinse off unattached virus particles. Subsequently, the cells were cultured in fresh medium for different times for the detection of autophagosome accumulation. NA cells treated with the autophagy inducer rapamycin were used as positive controls.

### Sample preparation and iTRAQ labeling

The brain tissues used for proteomics analysis were ground to a powder in liquid nitrogen and then lysed in lysis buffer (8 M urea, 4% 3-(3-cholamidopropyl)-dimethylammoniopropane sulfonate (CHAPS), and 25 mM Tris Base, pH 8.5; all purchased from BIO BASIC Inc., Ontario, Canada). After sonication for further lysis, the supernatant of the tissue lysate was treated with dithiothreitol (DTT) at 56°C for 1 h to denature the disulfide bonds. The samples were subsequently alkylated through incubation with iodoacetamide for 45 min in a dark chamber. The proteins were further purified through precipitation using acetone, and the pellets were redissolved in 0.5 M tetraethylammonium bromide (TEAB; Applied Biosystems, CA, USA). The protein concentration was determined using the 2-D Quan Kit (GE Healthcare Life Sciences, NJ, USA), and 100 μg of protein per sample was digested using sequence-grade modified trypsin (Promega; WI, USA) overnight at 37°C. The peptides were harvested and further labeled with iTRAQ reagents (iTRAQ 8plex Kit, AB SCIEX, MA, USA) according to the manufacturer's instructions. The labeled peptides were mixed and dried through vacuum centrifugation.

### Strong cationic exchange (SCX) fractionation and LC-MS/MS analysis

iTRAQ-labeled peptides were fractionated through SCX chromatography on an Ultremex SCX column using an LC-20AB high-performance liquid chromatography (HPLC) system (Shimadzu, Kyoto, Japan), and a total of 13 fractions were collected. Each fraction was desalted and dried for subsequent tandem MS analysis. All fractions were analyzed on a Q-Exactive mass spectrometer (Thermo Fisher Scientific, CA, USA) coupled to an LC-20AD nanoflow HPLC instrument (Shimadzu, Kyoto, Japan).

### Database searching

Tandem mass spectra were extracted, and the charge state was deconvoluted and deisotoped using Mascot Distiller version 2.5.1 (Matrix Science, London, UK). Mascot was set up to search the concatenated UniProt mouse proteome (201512, 16,734 entries) and the contaminants_20120713 database from the Max Planck Institute, assuming trypsin enzyme digestion. Mascot was searched using a fragment ion mass tolerance of 0.020 Da and a parent ion tolerance of 10.0 PPM. The carbamidomethyl of cysteine, the iTRAQ 8plex of lysine and the N terminus were specified in Mascot as fixed modifications. The oxidation of methionine, the acetyl of the N terminus and the iTRAQ 8plex of tyrosine were specified in Mascot as variable modifications.

### Criteria for protein identification

MS/MS-based peptide and protein identifications were validated with Scaffold (version Scaffold_4.4.8, Proteome Software Inc., Portland, OR, USA). The peptides were successfully identified when the Failure Discrepancy Report (FDR) was less than 0.01 based on a Scaffold Local FDR algorithm. Protein identifications were accepted when an FDR value less than 0.01 was achieved and at least two peptides were identified. The protein probabilities were assigned using the Protein Prophet algorithm [53]. Proteins containing similar peptides that could not be differentiated based on an MS/MS analysis alone were grouped to satisfy the principles of parsimony.

### Quantitative data analysis

Peptide quantitation and protein identification were performed using Scaffold Q+ (version Scaffold_4.4.8, Proteome Software Inc., Portland, OR, USA). Two individual datasets were merged using the Scaffold Q+ algorithm. The peptide identification intensities were normalized within the assigned protein. The reference channels were normalized to produce a 1:1 FC. All normalization calculations were performed using medians to multiplicatively normalize the data. Proteins were considered DEPs when the |FC| was higher than 1.3 according to a permutation test at p≤0.05.

### Transmission electron microscopy

TEM was performed to observe the ultrastructures of autophagosome-like vesicles. Monolayer of NA cells were mock-infected, infected with RABV CVS-11 or SRV9 strain at a MOI of 5 or treated with 100 nM rapamycin (an inducer of autophagy; Sigma-Aldrich, MO, USA) as a positive control. The cells were washed with phosphate-buffered saline (PBS) at 36 hpi and collected by centrifugation at 1,000 rpm for 10 min. The cell mass was fixed in 4% glutaraldehyde. Ultrathin sections were examined with a Tecnai G2 Spirit TEM instrument (FEI, OR, USA). For quantitative analysis, at least 10 cells from each sample were selected, and the number of autophagosomes in each cell was counted by an observer blinded to the experimental conditions.

### Western blot analysis

Cells were cultured in six-well plates and washed with PBS at the indicated time points after viral infection or drug treatment. After harvesting, the cells were lysed in radioimmunoprecipitation assay buffer (RIPA; Beyotime Biotechnology, Jiangsu, China) containing 1 mM phenylmethylsulfonyl fluoride (PMSF; Beyotime Biotechnology, Jiangsu, China). The total protein was quantified using the bicinchoninic acid (BCA) method (Pierce, IL, USA). The lysates were boiled for 5 min in 5× reducing sodium dodecyl sulfate-polyacrylamide gel electrophoresis (SDS-PAGE) loading buffer. Equivalent masses of each sample were separated using 5-12% SDS-PAGE and wet-transferred to a 0.45-μm polyvinylidene fluoride (PVDF) membrane (Millipore, MA, USA). The membranes were blocked using SuperBlock Blocking Buffer in PBS (Pierce, IL, USA) containing 0.5% Tween 20 for 1 h at room temperature and subsequently probed overnight with LC3B antibodies as a marker of autophagy (Sigma-Aldrich, MO, USA) at 4°C, and anti-β-tubulin antibodies (Beijing Ray Antibody Biotech, Beijing, China) were used as a protein loading control. After washing with PBST, the membranes were incubated with horseradish peroxidase (HRP)-conjugated goat anti-rabbit IgG (Santa Cruz, CA, USA) or goat anti-mouse IgG (Bioworld Technology, MN, USA) at room temperature for 1 h. The signals were obtained on a Fujifilm LAS-4000 image reader (Fujifilm, Tokyo, Japan) with the SuperSignal West Dura Extended Duration Substrate Kit (Pierce, IL, USA), and quantification of the protein blots was performed using Multi Gauge software (version 3.1).

## SUPPLEMENTARY MATERIALS FIGURES AND TABLES










