# Supplementary material for: Autophagy is highly targeted among host comparative proteomes during infection with different virulent RABV strains
Source: Oncotarget. 2017 Feb 8;8(13):21336–50. doi: 10.18632/oncotarget.15184 (PMC5400588; doi:10.18632/oncotarget.15184)
Supplement: Supplementary file 1 [file oncotarget-08-21336-s001.pdf]

Autophagy is highly targeted among host comparative proteomes during infection with different virulent RABV strains

SUPPLEMENTARY DATA

The proteomic analysis showed significantly different degrees of ROS induction between CVS-11 and SRV9 infection. To validate this result, the generation of ROS was measured using the fluorescent dye 6-carboxy-2′, 7′-dichlorodihydrofluorescein diacetate (DCFH-DA, Beyotime Biotechnology, Jiangsu, China) and observed with a FACSCalibur flow cytometer (Becton Dickinson Immuno-cytometry System, CA, USA). As shown in the Supplementary Figure 1, the levels of ROS generation in the RABV- and mock-infected NA cells were indistinct at 12 and 24 hpi. An increased production of ROS was observed at 36 hpi in the CVS-11 group, and this level of ROS generation was maintained through 48 hpi. In comparison, the SRV9-induced increase in ROS generation was delayed to 48 hpi.

mTOR is an important signaling molecule that regulates diverse cellular process, including autophagy. Activation of mTOR acts as a negative regulator of autophagy [1, 2]. A bioinformatics analysis showed that the mTOR pathway is significantly different in NA cells infected with CVS-11 compared with NA cells infected with SRV9. To validate this result, the phospho-mTOR and total mTOR levels were analyzed by western blotting. As shown in the Supplementary Figure 2, the total mTOR level in CVS-11-infected NA cells was decreased compared with those in SRV9- and mock-infected NA cells, which is consistent with the results of the iTRAQ-based analysis. However, the phospho-mTOR level was decreased in SRV9- but not CVS-11-infected NA cells, implying that mTOR signaling is suppressed during SRV9 infection. It has been demonstrated that the class I PI3K/Akt/mTOR/p70S6K pathway is involved in autophagy during exposure to certain conditions [3]. Therefore, we examined the phosphorylation levels of Akt and p70S6K and found that these were not altered upon RABV infection, indicating that Akt and p70S6K are not the upstream modulator and downstream sensors of mTOR, respectively, in RABV-induced autophagy. A recent study demonstrated that AMPK is the upstream regulator of wide-type RABV-induced autophagy [4].

REFERENCES

1. Shrivastava S, Bhanja Chowdhury J, Steele R, Ray R, Ray RB. Hepatitis C virus upregulates Beclin1 for induction of autophagy and activates mTOR signaling. J Virol. 2012; 86:8705-8712.
2. He C, Klionsky DJ. Regulation mechanisms and signaling pathways of autophagy. Annu Rev Genet. 2009; 43:67-93.
3. Levine B, Kroemer G. Autophagy in the pathogenesis of disease. Cell. 2008; 132:27-42.
4. Peng J, Zhu S, Hu L, Ye P, Wang Y, Tian Q, Mei M, Chen H, Guo X. Wild-type rabies virus induces autophagy in human and mouse neuroblastoma cell lines. Autophagy. 2016; 12:1704-1720.

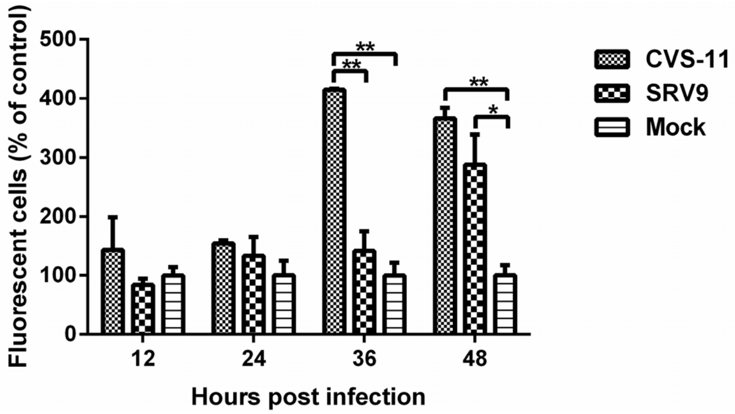

Supplementary Figure 1: Different virulent RABV strains have dissimilar characteristics in inducing the production of ROS in NA cells. Monolayer of NA cells were mock-infected or infected with the RABV CVS-11 or SRV9 strain at a MOI of 5 for the indicated time, exposed to DCFH-DA, and subjected to flow cytometry analysis. The ratio of fluorescent cells compared with control are shown. *$p \leq 0.05$, **$p \leq 0.01$, ***$p \leq 0.001$ vs. the mock-infected group.

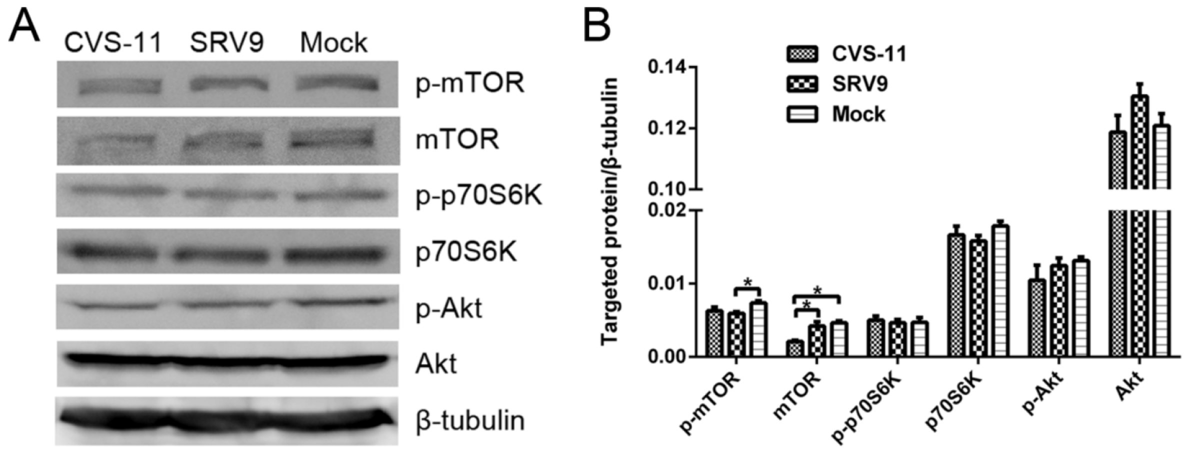

Supplementary Figure 2: Signaling pathways involved in NA cells infected with RABV. A. The cells were mock-infected or infected with CVS-11 or SRV9 at a MOI of 5 for 36 h, and the cell lysates were analyzed for the activation of mTOR, p70S6K and Akt. Total antibodies against mTOR (cat#2972), p70S6K (cat#9202), and Akt (cat#9272) and phospho-specific antibodies against mTOR (Ser2448, cat#5536), p70S6K (Thr389, cat#9205) and Akt (Ser473, cat#4060) were obtained from Cell Signaling Technology (MA, USA). Representative images from three replicates are shown. **B.** The intensity ratio of targeted proteins to β-tubulin was calculated. The data represent the mean ± SD of 3 independent experiments.

Supplementary Table 1: Complete list of the dysregulated proteins identified using the protein profile of the mock-infected mouse brain as the background

See Supplementary File 1

Supplementary Table 2: Diseases and biological functions identified at three time points

See Supplementary File 2

Supplementary Table 3: The list of identified networks

See Supplementary File 3

Supplementary Table 4: The details of the dysregulated pathways

See Supplementary File 4
